# The Toll-like Receptor 7-Mediated Ro52 Antigen-Presenting Pathway in the Salivary Gland Epithelial Cells of Sjögren’s Syndrome

**DOI:** 10.3390/jcm12134423

**Published:** 2023-06-30

**Authors:** Shin-Ya Nishihata, Toshimasa Shimizu, Masataka Umeda, Kaori Furukawa, Kaname Ohyama, Atsushi Kawakami, Hideki Nakamura

**Affiliations:** 1 Department of Immunology and Rheumatology, Division of Advanced Preventive Medical Sciences, Nagasaki University Graduate School of Biomedical Sciences, Nagasaki 852-8501, Japan; nissyhatter24@gmail.com (S.-Y.N.); toshimasashimizu2000@yahoo.co.jp (T.S.); masatakau0807@nagasaki-u.ac.jp (M.U.); furukaka@nagasaki-u.ac.jp (K.F.); atsushik@nagasaki-u.ac.jp (A.K.); 2 Department of Molecular Pathochemistry, Graduate School of Biomedical Sciences, Nagasaki University, Nagasaki 852-8501, Japan; k-ohyama@nagasaki-u.ac.jp; 3 Division of Hematology and Rheumatology, Department of Medicine, Nihon University School of Medicine, Tokyo 113-8602, Japan

**Keywords:** TLR7, MHC class I, Ro52, Sjögren’s syndrome

## Abstract

Objective: To investigate whether stimulation with toll-like receptor (TLR) 7 leads to pathways that proceed to tripartite motif-containing protein 21 (TRIM21) or Ro52/SS-A antigen presentation through major histocompatibility complex (MHC) class I in salivary gland epithelial cells (SGECs) from Sjögren’s syndrome (SS) patients. Design and Methods: Cultured SGECs from SS patients were stimulated with TLR7 agonist, loxoribine, and interferon-β. Cell lysates immunoprecipitated by anti-MHC class I antibody were analyzed by Western blotting. The immunofluorescence of salivary gland tissue from SS and non-SS subjects and cultured TLR7-stimulated SGECs was examined. Results: Significantly increased MHC class I expression was observed in SS patients’ ducts versus non-SS ducts; no significant difference was detected for ubiquitin. Upregulated MHC class I in the cell membrane and cytoplasm and augmented Ro52 expression were observed in SGECs stimulated with TLR7. The formation of peptide-loading complex (PLC), including tapasin, calreticulin, transporter associated with antigen processing 1, and endoplasmic reticulum-resident protein 57 in labial salivary glands (LSGs) from SS patients, was dominantly observed and colocalized with MHC class I, which was confirmed in TLR7-stimulated SGEC samples. Conclusion: These findings suggest that the TLR7 stimulation of SS patients’ SGECs advances the process toward the antigen presentation of TRIM21/Ro52-SS-A via MHC class I.

## 1. Introduction

Sjögren’s syndrome (SS) is a systemic autoimmune disease that mainly affects exocrine glands such as the salivary and lacrimal glands [1,2,3]. The pathogenesis of SS is still unclear. The involvement of genetic and environmental factors induces immune abnormalities and is intricately involved in the pathogenesis and control of the disease [4,5]. Various autoantibodies such as anti-Ro/SS-A and anti-La/SS-B antibodies are produced in SS [6,7], and the pathogenesis of SS was thought to center on immune abnormalities according to the activation of acquired immunity and tissue damage caused by the infiltration of mononuclear cells, mainly CD4-positive T cells [8]. However, it has been observed that innate immune responses that activate acquired immunity and induce inflammation are also important in the pathogenesis and control of SS [9,10,11].

The innate immune response is a defense mechanism that rapidly induces inflammation and the immune response upon the recognition of pathogens. Pattern recognition receptors (PRRs) are the initiators of immune responses, and PRRs recognize pathogen-associated molecular patterns (PAMPs), which are molecular structures derived from foreign microorganisms [12]. Toll-like receptor (TLR) 7, a PRR, localizes within the endoplasmic reticulum and endosomes, and TLR induces the production of type I interferon by recognizing nucleic acids [13]. TLR7 is also expressed in the ductal epithelial cells of individuals with SS, and our research has demonstrated that TLR7 stimulation enhances downstream signaling on salivary gland tissues and cells from patients with SS [14,15].

We have also observed increased expressions of Ro52 and MHC class I in salivary gland epithelial cells (SGECs) from patients with SS upon TLR7 stimulation in vitro [14]. Ro52 is a member of the tripartite motif protein (TRIM) family, and is designated TRIM21 [16,17]. It is also an E3 ubiquitin ligase involved in immune host defense, and a target of autoantibodies in autoimmune diseases such as SS [18,19].

MHC class I molecules are present on all nucleated cells and present antigenic peptides to cytotoxic T lymphocytes (CTLs) [20]. As a first step of antigen presentation by MHC class I, endogenous antigens are ubiquitinated and then deubiquitinated by proteasomes and degraded to peptides [21]. The degraded peptides move into the interior of the rough endoplasmic reticulum via the transporter associated with antigen processing (TAP) and bind to MHC class I molecules by the action of a complex of TAP, tapasin, MHC class I, endoplasmic reticulum-resident protein 57 (ERp57), and calreticulin, which together are called the peptide-loading complex (PLC) [22]. The class I/antigen–peptide complex then passes through the Golgi apparatus and is presented to the T-cell receptors on CD8+ T cells [23].

We hypothesized that TLR7-stimulated SGECs may present the Ro52 antigen via MHC class I, which is also activated by TLR7 ligation. However, this pathway has not been elucidated in the pathogenesis of SS. In this study, we examined whether TLR7 stimulation advances the steps of the MHC class I-mediated Ro52 antigen presentation pathway in SGECs derived from SS patients.

## 2. Materials and Methods

### 2.1. Patients

For the immunofluorescence analysis, we retrospectively analyzed materials from 10 patients with primary SS and 5 control subjects who visited Nagasaki University Hospital during the period 2008–2022. The patients’ SS classification was based on the 2016 American College of Rheumatology (ACR)/European League Against Rheumatism (EULAR) classification criteria for primary Sjögren’s syndrome (SS) [24]. The anti-Ro/SS-A antibody-seronegative control subjects had sicca symptoms but did not fulfill the 2016 ACR/EULAR classification criteria (non-SS sicca control subjects). Labial salivary gland (LSG) biopsy specimens were obtained from all participants for our assessment of the pathological findings of SS. For the determination of focus scores (i.e., the number of foci per 4 mm^2^) in LSGs, the number of foci in a section from LSGs was counted and the surface area of the section was measured by a hybrid cell count system mounted on a microscope (BZ-X710; Keyence, Osaka, Japan).

The clinical and serological characteristics of the primary SS patients and control subjects are summarized in Table 1. All patients gave their informed consent to be subjected to the protocol, which was approved by the Institutional Ethics Committee of Nagasaki University Hospital (approval no. 20091410).

### 2.2. Culture of Salivary Gland Epithelial Cells (SGECs)

We performed the culturing of SGECs as described [25]. Briefly, the LSG tissues were cut with fine needles and scalpels and placed in six-well plates coated with type I collagen (Iwaki, Tokyo) with culture medium consisting of defined keratinocyte-SFM culture medium (Invitrogen Life Technologies, Carlsbad, CA, USA), 0.4 μg/mL hydrocortisone (Sigma-Aldrich, St. Louis, MO, USA), 25 μg/mL bovine pituitary extract (Kurabo, Osaka, Japan), 100 U/mL penicillin, and 100 μg/mL streptomycin (Gibco, Grand Island, NY, USA). When an outgrowth of SGECs was observed, the cells were transferred into 100 mm^2^ plates coated with type I collagen (Iwaki) after the cells reached confluence for the analysis by a Simple Western system.

When the SGECs reached confluence, the cells were treated with 1 mM loxoribine, a TLR7 ligand (InvivoGen, San Diego, CA, USA), for 6 h, and then with 1000 U/mL of interferon (IFN)-β (Betaferon^®^; Bayer Pharma, Berlin, Germany) for 12 h as described [14]. For immunofluorescence, SGECs were distributed onto 12 mm^2^ cover slips coated with a type I collagen, Cellmatrix (Nitta Gelatin, Osaka, Japan) in 24-well plates (Corning, New York, NY, USA) after the SGECs reached confluence on 100 mm^2^ plates. Subsequently, the SGECs were treated with 1 mM loxoribine for 6 h and 1000 U/mL of IFN-β for 12 h.

### 2.3. Immunofluorescence

We performed an immunofluorescence examination to determine the localizations of MHC class I, Ro52, Ro60, ubiquitin, and peptide-loading complex (PLC, i.e., TAP1, tapasin, ERp57, and calreticulin) in LSGs in vivo and the expressions of MHC class I, Ro52, Ro60, and PLC in SGECs in vitro. A cancer tissue array including four types of cancer (colon, breast, lung, and prostate) and normal tissue (US Biomax, Derwood, MD, USA) was used as the positive control for class I and PLC expression (Supplementary Materials Figure S1). The primary antibodies (Supplementary Materials Table S1) used were rabbit anti-MHC class I polyclonal (Proteintech, Rosemont, IL, USA), mouse anti-MHC class I monoclonal (Novus Biologicals, Littleton, CO, USA), rabbit anti-Ro52 polyclonal (Cloud-Clone Corp.; Katy, TX, USA), mouse anti-Ro60 monoclonal (Santa Cruz, Dallas, TX, USA), mouse anti-ubiquitin monoclonal (Enzo Life Sciences, Farmingdale, NY, USA), rabbit anti-TAP1 monoclonal (Bioss Antibodies, Woburn, MA, USA), rabbit anti-tapasin polyclonal (GeneTex, Irvine, CA, USA), mouse anti-ERp57 monoclonal (Boster Bio, Pleasanton, CA, USA), and rabbit anti-calreticulin polyclonal antibody (LifeSpan BioSciences, Seattle, WA, USA).

Briefly, paraffin-embedded sections from LSGs and the tissue array were incubated with 3% H_2_O_2_ solution for the inhibition of endogenous peroxidase activity after microwave epitope retrieval, and blocked with 5% normal horse serum. They were incubated with each primary antibody diluted with 5% normal horse serum at 4 °C overnight. After incubation with primary antibodies, the sections from LSGs were reacted with secondary antibodies, including donkey anti-mouse IgG conjugated with fluorescein isothiocyanate (FITC) antibody (Jackson ImmunoResearch Laboratories, West Grove, PA, USA), donkey anti-rabbit IgG conjugated with tetramethyl rhodamine isothiocyanate (TRITC) antibody (Jackson ImmunoResearch Laboratories), donkey anti-goat IgG conjugated with FITC antibody (Jackson ImmunoResearch Laboratories), and Hoechst dye 33,258 (Sigma-Aldrich), for 45 min at room temperature (RT) in the dark. The sections were then mounted in Vectashield mounting medium (Vector Laboratories, Burlingame, CA, USA).

In vitro, SGECs on 12 mm^2^ cover slips were incubated in 4% paraformaldehyde for 10 min at 4 °C, and then immersed in methanol for 10 min at −20 °C after loxoribine and IFN-β stimulation. The cells were blocked with 5% normal horse serum and incubated in each primary antibody for 60 min at RT. The primary antibodies used were rabbit anti-MHC class I polyclonal (Proteintech), mouse anti-MHC class I monoclonal (Novus Biologicals), rabbit anti-Ro52 polyclonal (Cloud-Clone Corp.), mouse anti-Ro60 monoclonal (Santa Cruz), rabbit anti-TAP1 polyclonal (Bioss Antibodies), rabbit anti-tapasin polyclonal (GeneTex), mouse anti-ERp57 monoclonal (Boster Bio), and goat anti-calreticulin polyclonal antibody (LifeSpan BioSciences).

After incubation with primary antibodies, the SGECs were reacted with secondary antibodies, including donkey anti-mouse IgG conjugated with FITC antibody (Jackson ImmunoResearch Laboratories), donkey anti-rabbit IgG conjugated with TRITC antibody (Jackson ImmunoResearch Laboratories), donkey anti-goat IgG conjugated with FITC antibody (Jackson ImmunoResearch Laboratories), and Hoechst dye 33,258 (Sigma-Aldrich) for 45 min at RT in the dark. The cells were subsequently mounted in Vectashield mounting medium. Images were captured by a microscope (BZ-X710). The mean fluorescence intensity (MFI) of cells in a given area was calculated by the hybrid cell count system that was mounted on the BZ-X710 microscope.

### 2.4. Deconvolution Technique

High-resolution images of sections of the LSGs and SGECs were obtained by a deconvolution system installed in the BZ-X710 microscope.

### 2.5. Simple Western Analysis, Coimmunoprecipitation (co-IP)

We performed a Simple Western analysis to examine MHC class I binding to Ro52 after stimulation with loxoribine and IFN-β. For coimmunoprecipitation (co-IP), after SGECs were lysed and the protein concentrations were measured, identical amounts of protein and HepG2 cell lysate (Santa Cruz) as the positive control were cleared and incubated with 25 μL of Protein G Sepharose™ 4 Fast Flow (Cytiva, Tokyo, Japan) and 5 μg of primary antibodies overnight at 4 °C. The primary antibodies used were rabbit anti-MHC class I polyclonal (Proteintech) and Normal rabbit IgG (Medical & Biological Laboratories, Nagoya, Japan) as the negative control. Co-IP samples were mixed with 30 μL of fluorescent 1× Master Mix containing 200 mM dithiothreitol (ProteinSimple, Bio-Techne, San Jose, CA, USA) and denatured at 95 °C for 5 min.

The primary antibodies used were rabbit anti-MHC class I polyclonal (Proteintech), rabbit anti-Ro52 polyclonal (Cloud-Clone), and mouse anti-Ro60 monoclonal (Santa Cruz). Mouse anti-rabbit IgG light chain coupled to horseradish peroxidase (HRP) monoclonal antibody (working dilution 1:100; Abcam, Cambridge, MA, USA) and goat anti-mouse IgG light chain coupled to HRP polyclonal antibody (working dilution 1:100; Jackson ImmunoResearch Laboratories) that were employed to avoid the detection of heavy chains were used as secondary antibodies. These antibodies were diluted in Antibody Diluent 2 (ProteinSimple).

The prepared samples, biotinylated ladder, primary antibodies, secondary antibodies, and chemiluminescent substrate were added to the designated wells in the assay plate. The prepared assay plate was placed in the Simple Western (ProteinSimple) machine [26], followed by the addition of Simple Western assay buffers in the system tray and the insertion of capillaries. The injection volume for each sample was 4 μL. All subsequent separation, immunodetection, and analysis steps were performed automatically by the instrument. Compass software (ver. 5.0.1, Atlanta, GA, USA) was used to visualize the Simple Western lanes.

### 2.6. Statistical Analysis

We used the Mann–Whitney U test or Fisher’s exact test to compare clinical and serological characteristics, and Welch’s t-test to compare the MFI of protein expression between the SS and control groups. All statistical analyses were performed using JMP software, ver. 17 (SAS, Cary, NC, USA) and GraphPad prism (ver. 9.5.1, GraphPad Software, La Jolla, CA, USA). *p*-values < 0.05 were considered significant.

## 3. Results

### 3.1. Subject Characteristics

Table 1 summarizes the characteristics of the 10 patients with SS and the 5 control subjects. All of the SS patients were female. Compared to the control group, the SS group had significantly greater anti-SS-A/Ro antibody, antinuclear antibody, and rheumatoid factor positivity and significantly higher serum IgG levels and LSG biopsy focus scores (*p* < 0.001, *p* < 0.01, *p* < 0.01, *p* = 0.014, and *p* = 0.002, respectively).

### 3.2. Increased Expression of MHC Class I and Varying Expression of Ubiquitin in the Ducts from SS Patients’ LSGs

**Figure 1:**
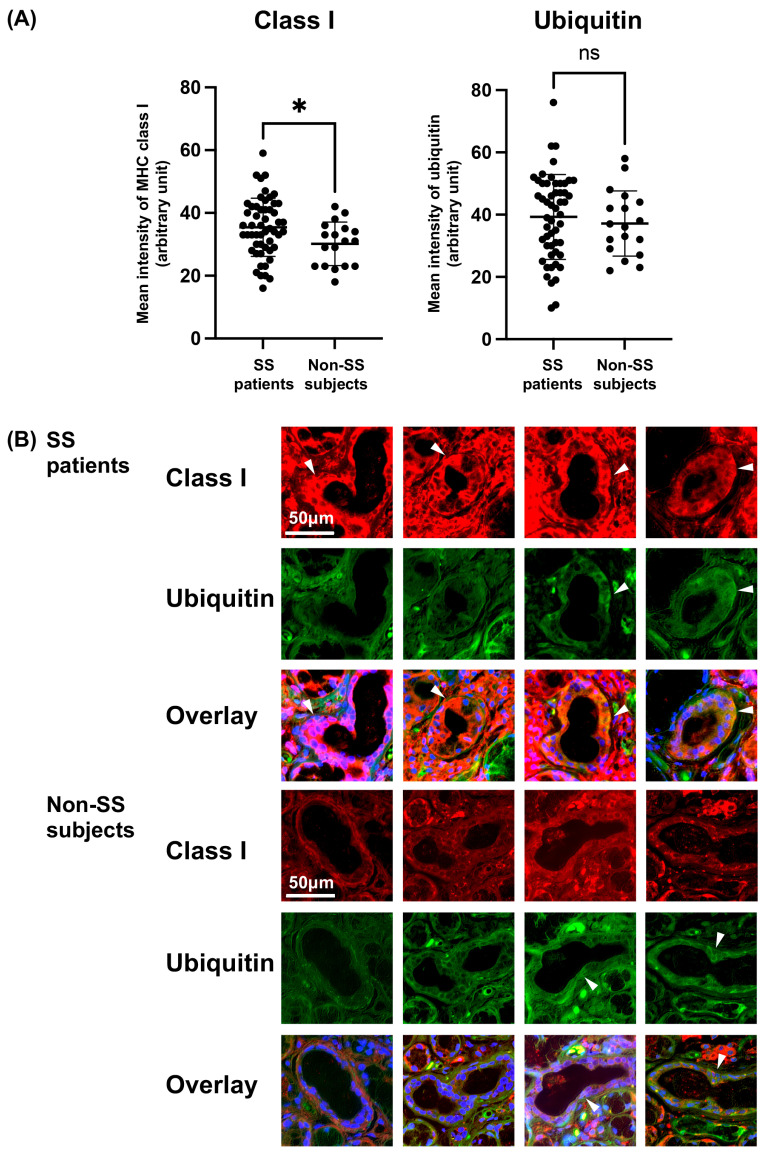
Increased expression of MHC class I and various expressions of ubiquitin in the ducts from SS patients’ LSGs. (**A**) The mean fluorescence intensity (MFI) of MHC class I and ubiquitin on the ducts of labial salivary glands (LSGs) from patients with Sjögren’s syndrome (SS) (total 53 ducts from 10 patients) and non-SS subjects’ ducts (total 18 ducts from 5 subjects). The MFI of immunostaining was captured and calculated with a hybrid cell count system. Significance was determined using Welch’s *t*-test. * *p* < 0.05, NS: not significant. (**B**) Representative samples of LSGs from SS patients (*n* = 10) and non-SS subjects (*n* = 5) stained with anti-class I (red) and anti-ubiquitin (green) antibodies. Hoechst (blue) was used for counterstaining the nuclei. White arrowheads: the identical ductal expression of staining for different proteins. Bar: 50 μM. Non-SS: these subjects were classified as non-SS sicca control subjects based on the AECG classification criteria.

We examined the expression of MHC class I and ubiquitin in ducts of LSGs from the primary SS patients and non-SS subjects. The MFI of MHC class I was significantly higher in the ducts of LSGs from the primary SS patients (Figure 1A), but the MFI of ubiquitin was not significant (Figure 1A) between the SS and non-SS subjects.

The expression of MHC class I was increased in the ducts of LSGs from the SS patients compared to the non-SS subjects, while similar expressions of ubiquitin were observed in the ducts of LSGs from both the SS and non-SS subjects (Figure 1B).

### 3.3. Increased Expression of MHC Class I and Ro52 in TLR7-Stimulated SGECs

**Figure 2:**
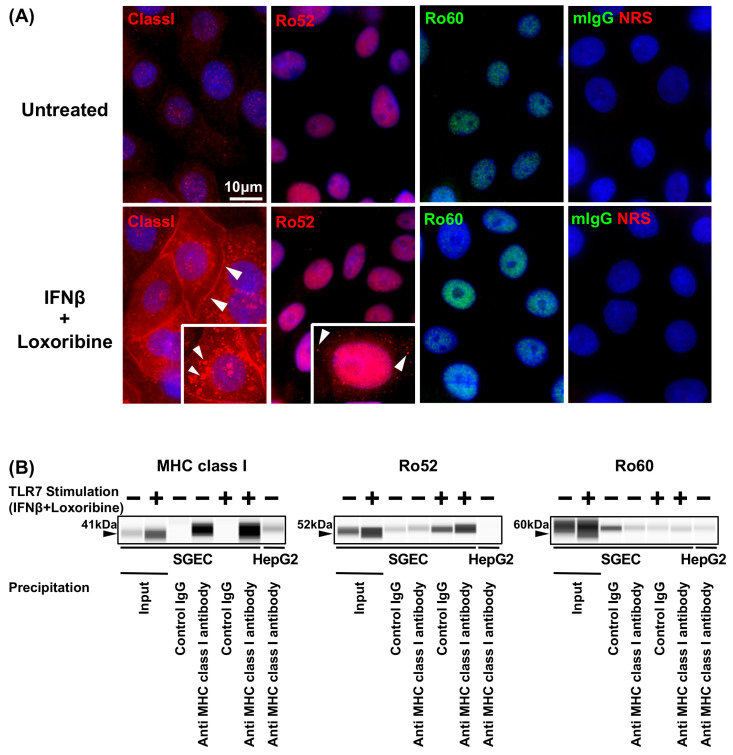
Increased expression of MHC class I and Ro52 in TLR7-stimulated SGECs. (**A**) Representative images in immunostaining showing the expressions of MHC class I, Ro52 (red), and Ro60 (green) in SGECs from SS patients (*n* = 2) stimulated with 1 mM loxoribine for 6 h and 1000 U/mL of IFN-β for 12 h. mIgG1 (green) and NRS (red) were used as isotype controls. White arrowheads: the expression of cytoplasm. Bar: 10 μM. mIgG: mouse IgG, NRS: normal rabbit serum. (**B**) MHC class I and Ro52 and Ro60 signals in SGECs immunoprecipitated by control rabbit IgG or rabbit anti MHC class I antibody from SS patients (*n* = 4) stimulated with 1 mM loxoribine for 6 h and/or 1000 U/mL of IFN-β for 12 h analyzed by a Simple Western system. One representative blot is shown. Schemes follow the same formatting.

MHC class I was strongly expressed in the cytoplasm and plasma membrane in SGECs after stimulation with the TLR7 ligand. The punctiform expression of Ro52 was detected in cytoplasm SGECs after stimulation with the TLR7 ligand. In contrast, the expression of Ro60 was not changed in SGECs after stimulation with the TLR7 ligand (Figure 2A).

The expression level of Ro52 immunoprecipitated with anti-MHC class I antibodies in SGECs as well as HepG2 as the positive control was increased in SGECs after stimulation with the TLR7 ligand (Figure 2B and Figure S2). In contrast, the expression levels of Ro60 immunoprecipitated with anti-MHC class I antibodies were not increased in SGECs after stimulation with the TLR7 ligand (Figure 2B and Figure S2).

### 3.4. Increased Expression of PLC and Class I in SS Patients’ LSGs

**Figure 3:**
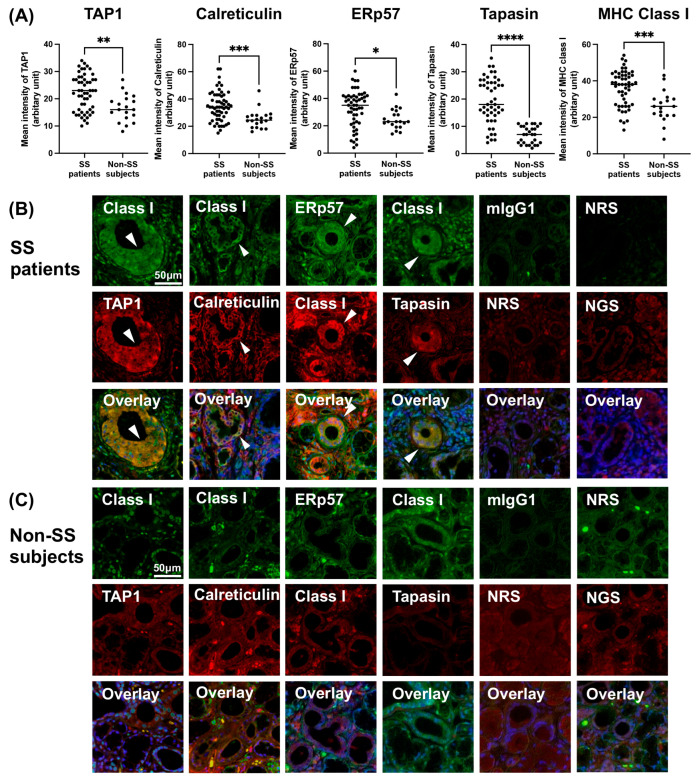
Increased expression of PLC and class I in SS patients’ LSGs. (**A**) The mean fluorescence intensity (MFI) of MHC class I (total 53 ducts from 10 SS, 19 ducts from 5 non-SS), TAP1 (53 ducts from 10 SS, 19 ducts from 5 non-SS), calreticulin (53 ducts from 10 SS, 20 ducts from 4 non-SS), ERp57 (54 ducts from 10 SS, 20 ducts from 5 non-SS) and tapasin (48 ducts from 10 SS, 28 ducts from 5 non-SS) on the ducts of labial salivary glands (LSGs) from patients with SS and non-SS subjects’ ducts. The MFI of immunostaining was captured and calculated with a hybrid cell count system. Representative samples of LSGs from SS patients (**B**) and non-SS subjects (**C**) stained with anti-class I, anti-TAP1, anti-calreticulin, anti-ERp57, anti-tapasin antibodies. mIgG1 (green), NRS (red), and NGS (red) were used as isotype controls. Hoechst was used for counterstaining the nuclei. White arrowheads: the identical ductal expression of staining for different proteins. Bar: 50 μM. Non-SS: these subjects were classified as non-SS sicca control subjects based on the AECG classification criteria. TAP1: transporter associated with antigen processing 1; ERp57: endoplasmic reticulum-resident protein 57; mIgG: mouse IgG; NRS: normal rabbit serum; NGS: normal goat serum. Significance was determined using Welch’s *t*-test. * *p* < 0.05, ** *p* < 0.01, *** *p* < 0.001, **** *p* < 0.0001.

The expression of MHC class I and proteins constituting the PLC was stronger in the ducts of LSGs from the SS patients compared to those of the non-SS subjects (Figure 3A–C). MHC class I was colocalized with proteins constituting the PLC in the ducts of LSGs from the SS patients (Figure 3B). However, no obvious coexpression of MHC class I and PLC components was observed in the non-SS subjects’ LSG ducts (Figure 3C).

### 3.5. Increased Expression of PLC and Class I in SS Patients’ TLR7-Stimulated SGECs

**Figure 4:**
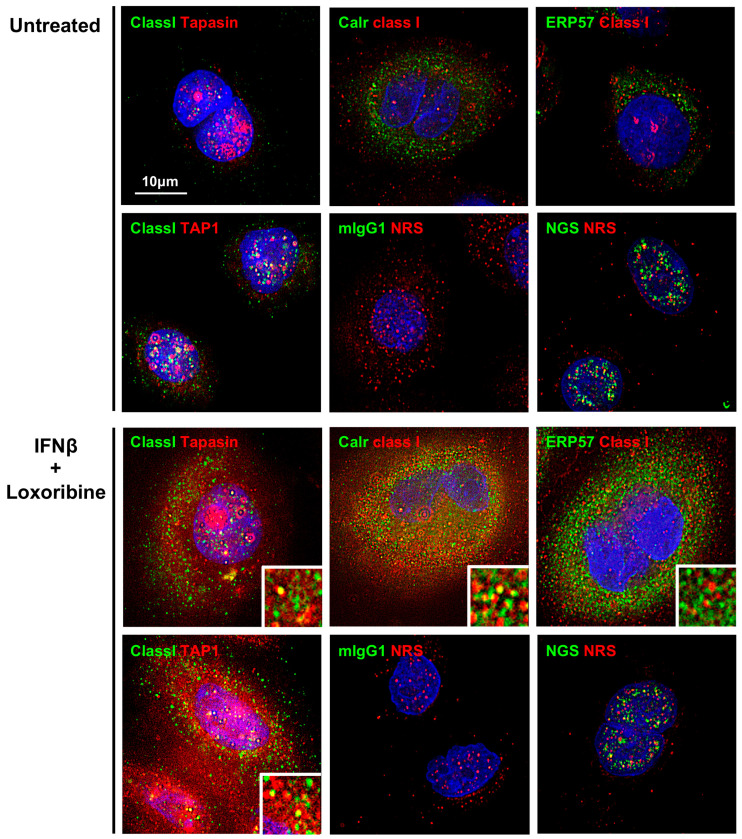
Increased expression of PLC and class I in SS patients’ TLR7-stimulated SGECs. Representative images in immunostaining showing the expressions of MHC class I, TAP1, calreticulin, ERp57, and tapasin in SGECs from SS patients (*n* = 2) stimulated with 1 mM loxoribine for 6 h and 1000 U/mL of IFN-β for 12 h. mIgG1 (green), NRS (red), and NGS (green) were used as isotype controls. Deconvolution was performed for all images in Figure 4. The *insets* show a magnified view of each panel. Bar: 10 μM. TAP1: transporter associated with antigen processing 1, ERp57: endoplasmic reticulum-resident protein 57, mIgG: mouse IgG, NRS: normal rabbit serum, NGS: normal goat serum.

Proteins constituting the PLC were strongly expressed in the cytoplasm of SGECs after stimulation with the TLR7 ligand. MHC class I was partly colocalized with proteins including tapasin, calreticulin, and TAP1 in the cytoplasm of SGECs after stimulation with the TLR7 ligand (Figure 4). No colocalization of MHC class I and ERp57 was observed.

## 4. Discussion

In the present in vivo analysis, compared to the non-SS group, ubiquitin on the ducts of LSGs from patients with SS showed different expression patterns, and MHC class I and proteins constituting the PLC showed increased expression. In vitro, the TLR7-stimulated SGECs from the primary SS patients also showed an increased expression of MHC class I on the cell membrane and cytoplasm, Ro52, and the components of the PLC.

Ro52 is one of the autoantigens of SS, and it plays a role in host immune defense and signal transduction as an E3 ubiquitin ligase [16]. Ro52 is also a target of autoantibodies in several autoimmune diseases, including SS [18]. Previously, TLR7-mediated stimulation using imiquimod was reported to enrich the protein level of Ro52 in HeLa cells [27]. In the present study, we observed that Ro52 was enhanced in the cytoplasm of TLR7-stimulated SGECs. It has been reported that TLR3/4 ligands upregulate the expression of Ro52 in macrophages and that TLR3 stimulation promotes Ro52 synthesis in SGECs, partially through the type I interferon pathway [28,29].

MHC class I also showed increased expression in salivary glands and SGECs stimulated with TLR7 in the present investigation. Wu et al. reported that the use of TLR2 agonist-fused protein increased the antigen presentation by MHC class I and induced cytotoxic T-lymphocyte responses [30]. Another study reported that MHC class I was upregulated in muscle cells by the administration of TLR7/8 agonist R-848 in autoimmune myositis, suggesting that TLR7 signaling is involved in Ro52 and MHC class I expression (except in immune cells) [31].

We also observed that Ro52 was upregulated in samples immunoprecipitated with MHC class I from TLR7-stimulated SGEC lysates. These results suggest that a TLR7-stimulated association of MHC class I and Ro52 may be involved in the antigen presentation of Ro52, an autoantigen. However, self-antigens must be cleaved to the size of antigen peptides in order to be recognized by the MHC class I groove. It is therefore possible that Ro52 not only associates with MHC class I but also undergoes a process of subsequent degradation into peptides.

One of the key steps in MHC class I antigen presentation is the ubiquitin–proteasome system. Endogenous antigens, which are the source of MHC class I ligands, are ubiquitinated by ubiquitin ligases, followed by deubiquitination and degradation to peptides by proteasomes [21]. The abundance of E3 ubiquitin ligases corresponds to the complex machinery of the proteome. As mentioned above, Ro52 is one of the E3 ubiquitin ligases involved in the ubiquitination of various proteins and has been shown to be involved in antigen presentation mechanisms. On the other hand, an earlier study reported that when antibody-bound pathogens enter the cytoplasm during infection, Ro52, an E3 ubiquitin ligase, recognizes IgG Fc and initiates self-ubiquitination, thereby directing pathogens to proteasomes for degradation. This mechanism suggests that Ro52 itself can be a candidate antigenic peptide [16].

In this study, we observed sites of both increased and decreased ubiquitin expression in response to MHC class I expression in the ducts of labial salivary glands from SS patients. The intensity of MHC class I expression in the salivary gland ducts of patients with SS was significantly higher than that observed in the ducts of non-SS patients. However, no significant difference in ubiquitin fluorescence intensity was observed between the ducts of the SS and non-SS subjects. The plots of ubiquitin fluorescence intensity for the SS patients exhibit large deviation. In other words, the plots demonstrate significant variation in the expression of ubiquitin within the ducts, suggesting that various stages of ubiquitination occur in vivo. These findings may be due to the different stages of polyubiquitination and deubiquitination in the ubiquitin–proteasome system for MHC class I-mediated antigen presentation.

Target antigen peptides degraded by the ubiquitin–proteasome system are loaded into MHC class I by the PLC in the endoplasmic reticulum in the cytoplasm [21,32]. Our present analyses revealed an increased expression of the PLC in ducts of labial salivary glands from primary SS patients compared to non-SS controls, as well as in SGECs stimulated with TLR7. This suggests that after TLR7 stimulation, PLCs that associate with degraded peptides after deubiquitination for antigen presentation via MHC class I are formed in the cytoplasm of SGECs.

Although the above findings indicate the mechanism of Ro52 antigen presentation by TLR7 activation in SS salivary glands, it is necessary to address some study limitations. We analyzed only a partial step in the whole process of MHC class I-mediated antigen presentation. Further analyses should be performed to reveal the actual degradation of Ro52 to peptides and its presentation to cytotoxic T cells. Regarding PLC formation, we did not observe the coexpression of MHC class I and ERp57. These results might be caused by the induction of a low amount of TLR7 ligation-induced ERp57 proteins. Another study limitation is the small sample size used to confirm in vitro PLC formation.

In conclusion, our results demonstrate that TLR7 stimulation enhanced MHC class I, Ro52, and PLC component proteins in the cytoplasm and induced varying ubiquitin expression patterns in SS salivary glands. These findings suggest a mechanism for antigen presentation via MHC class I of the self-antigen Ro52 by a TLR7 signaling pathway. Although the stimulation with TLR7 and the subsequent type I IFN secretion have been shown to be important in the pathogenesis of SS, there is no evidence of the antigen presentation of an autoantigen by the stimulation of TLR7. There is also no prior report that Ro52 proceeds to antigen presentation in the ductal epithelium, and our present results are novel findings showing that a TLR7 signaling pathway is involved in the previously unidentified mechanism of autoantigen presentation in salivary glands of individuals with primary SS. To further investigate the details of this step, it would be desirable to search for Ro52 peptides that can bind MHC class I and determine whether there is activation of cytotoxic T cells. As part of our ongoing research endeavors, we plan to undertake coculture experiments involving T cells and TLR7-stimulated SGECs. The elucidation of these mechanisms will greatly contribute to the clarification of the pathogenesis of SS and the development of preventive and therapeutic strategies for this disease.

## Figures and Tables

**Table 1:** Background information of the enrolled subjects.

Variables	pSS (*n* = 10)	Controls (*n* = 5)	*p*-Value
Age, year, median (IQR)	46.5 (16–60)	52 (43–60)	0.67 ^a^
Female, *n*	10	5	1 ^b^
Xerostomia, *n*	6 (60%)	4 (80%)	0.6 ^b^
Xerophthalmia, *n*	7 (70%)	4 (80%)	1 ^b^
Schirmer test positivity, *n*	7/9 (77.8%)	2 (40%)	0.27 ^b^
Saxon test positivity, *n*	7/9 (77.8%)	2 (40%)	0.27 ^b^
Anti-SS-A/Ro antibody positivity, *n*	10 (100%)	0 (0%)	<0.001 ^b^
Anti-SS-B/La antibody positivity, *n*	5 (50%)	0 (0%)	0.1 ^b^
ANA positivity, *n*	10 (100%)	1 (20%)	0.004 ^b^
RF positivity, *n*	8/8 (100%)	1 (20%)	0.007 ^b^
Serum IgG, mg/dl, median (IQR)	2071 (1550–2550)	1037 (568–1498) *n* = 3	0.014 ^a^
LSG biopsy, focus score	4 (3–11)	0	0.002 ^a^
ESSDAI score, median (IQR)	4/9 (0–12)	NA	NA

ANA: antinuclear antibody; ESSDAI: European League Against Rheumatism Sjögren’s Syndrome Disease Activity Index; IgG: immunoglobulin G; IQR: interquartile range; LSG: labial salivary gland; NA: not assessed; pSS: primary Sjögren’s syndrome; RF: rheumatoid factor. ^a^ Mann–Whitney U test; ^b^ Fisher’s exact test. *p*-values < 0.05 were considered significant.

## Data Availability

The data presented in this study are available on request from the corresponding author.

## Supplementary Materials

The following supporting information can be downloaded at: https://www.mdpi.com/article/10.3390/jcm12134423/s1, Figure S1: Expression of PLC in the cancer tissue array (US Biomax) as the positive control stained with anti-class I, anti-TAP1, anti-Calreticulin, anti-ERp57, and anti-Tapasin antibody. mIgG1 (green), NRS (red), and NGS (green) were used as isotype control. Hoechst was used for counterstaining the nuclei. White arrowheads: the identical ductal expression of staining for different proteins. Bar: 200 μM. mIgG: mouse IgG, NRS: normal rabbit serum, NGS: normal goat serum. m: mouse; r: rabbit; g: goat; Figure S2: MHC class I and Ro52 and Ro60 signal/noise ratios in SGECs immunoprecipitated by control rabbit IgG or rabbit anti-MHC class I antibody from SS patients (*n* = 4) stimulated with 1 mM loxoribine for 6 h and/or 1000 U/mL of IFN-β for 12 h analyzed by a Simple Western system. S/N ratio: signal/noise ratio; Table S1: Primary antibodies used in the present experiments.
