# Supplementary material for: The Toll-like Receptor 7-Mediated Ro52 Antigen-Presenting Pathway in the Salivary Gland Epithelial Cells of Sjögren’s Syndrome
Source: J Clin Med. 2023 Jun 30;12(13):4423. doi: 10.3390/jcm12134423 (PMC10342814; doi:10.3390/jcm12134423)
Supplement: Supplementary file 1 [file jcm-12-04423-s001.zip › jcm-2449169-supplementary.pdf]

SUPPLEMENTARY MATERIAL

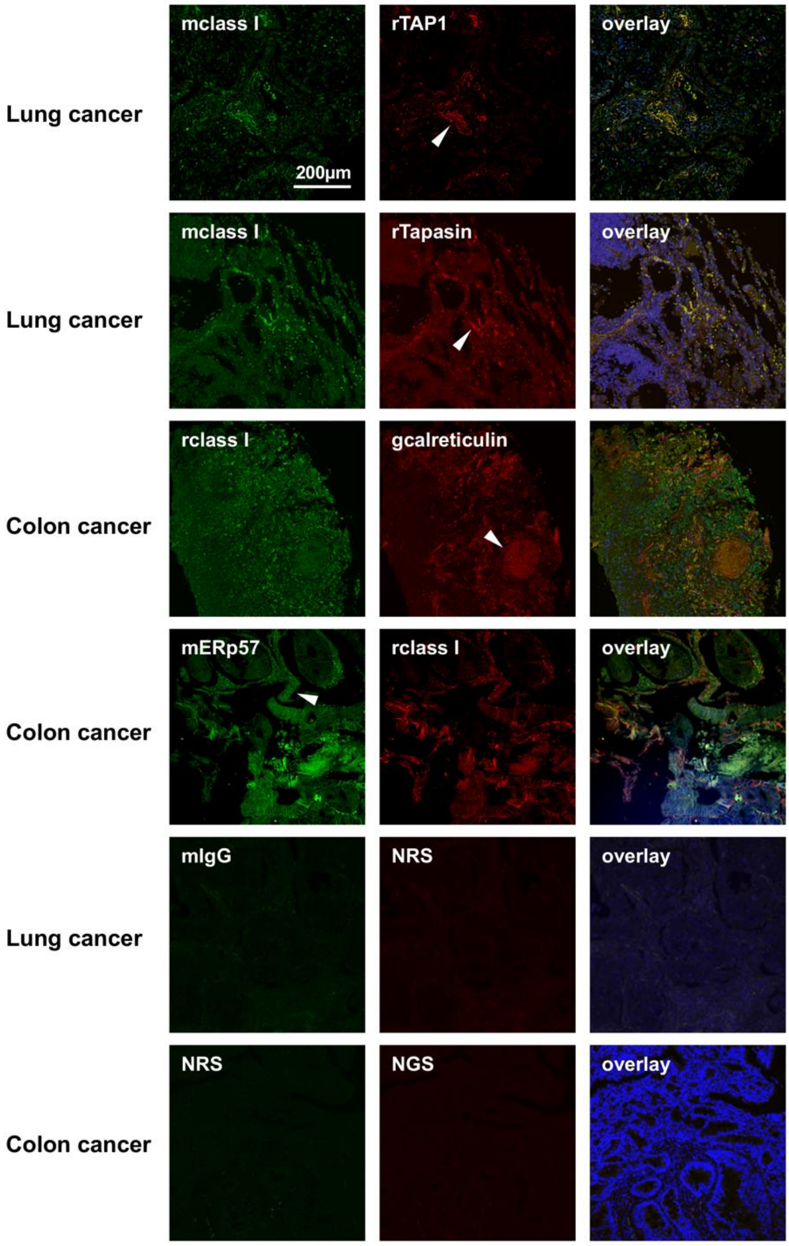

Figure S1. Expression of PLC in the cancer tissue array (US Biomax) as the positive control stained with anti-class I, anti-TAP1, anti-Calreticulin, anti-ERp57, and anti-Tapasin antibody. mIgG1 (green), NRS (red), and NGS (green) were used as isotype control. Hoechst was used for counterstaining the nuclei. *White arrowheads:* the identical ductal expression of staining for different proteins. Bar: 200 µM. mIgG: mouse IgG, NRS: normal rabbit serum, NGS: normal goat serum. m: mouse, r: rabbit, g: goat.

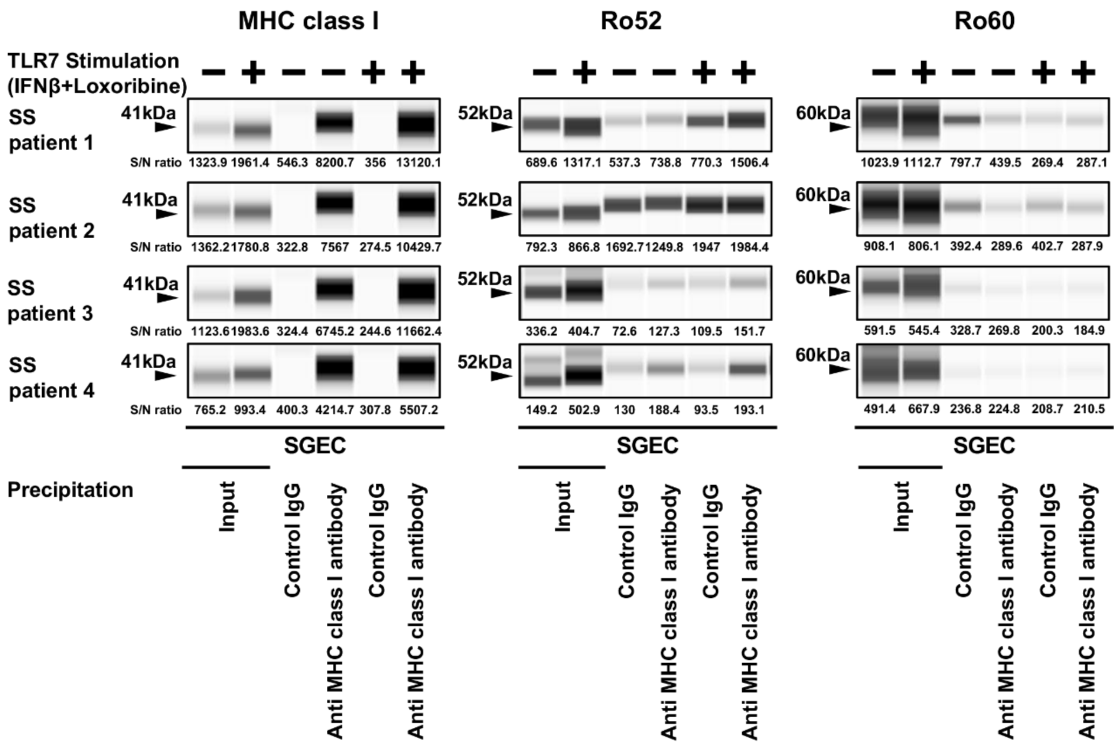

Figure S2. MHC class I and Ro52 and Ro60 signal/noise ratios in SGECs immunoprecipitated by control rabbit IgG or rabbit anti MHC class I antibody from SS patients (n=4) stimulated with 1 mM loxoribine for 6 hr and/or 1000 U/ml of IFN-β for 12 hr analyzed by a Simple Western system. S/N ratio: Signal/noise ratio.

Table S1. Primary antibodies used in the present experiments.

Antibodies	Immunogen	Poly/mono	Working dilution	Application	Source
Anti-MHC class I	HLA class I (HLA-A) fusion protein expressed in E.coli	Poly	1:100	IF	Rabbit
Same as above			1:20	Wes	
Anti-MHC class I	Rat MHC Class I	Mono	1:100	IF	Mouse
Anti-Ro52	SSA1 expressed in E.coli	Poly	1:100	IF	Rabbit
Same as above			1:20	Wes	
Anti-Ro60	Human 60-kDa Ro/SSA	Mono	1:100	IF	Mouse
Same as above			1:20	Wes	
Anti-ubiquitin	Poly-ubiquitinylated lysozyme.	Mono	1:250	IF	Mouse
Anti-TAP1	Human Tap1/ABCB2	Poly	1:100	IF	Rabbit
Anti-Tapasin	Human TAPBP	Poly	1:100	IF	Rabbit
Anti-ERp57	Human ERp57	Mono	1:100	IF	Mouse
Anti-Calreticulin	Human Calreticulin	Poly	1:100	IF	Goat

ABC: adenosine triphosphate (ATP)-binding cassette, ERp57: endoplasmic reticulum-resident protein 57, IF: immunofluorescence, Mono: monoclonal antibody, Poly: polyclonal antibody, TAP1: transporter associated with antigen processing 1, TAPBP: TAP-bindig protein, Wes: simple western analysis
